# Supplementary material for: Toxic Cyanopeptides Monitoring in Thermal Spring Water by Capillary Electrophoresis Tandem Mass Spectrometry
Source: Toxins (Basel). 2025 Jan 31;17(2):63. doi: 10.3390/toxins17020063 (PMC11860162; doi:10.3390/toxins17020063)
Supplement: Supplementary file 1 [file toxins-17-00063-s001.zip › toxins-3358624-supplementary.pdf]

Table S1. Analytes molecule.

Analyte	Formula	Molecular Mass (g/mol)	Structure	Contains the Adda residue?
NOD	$C_{41}H_{60}N_{8}O_{10}$	825.0	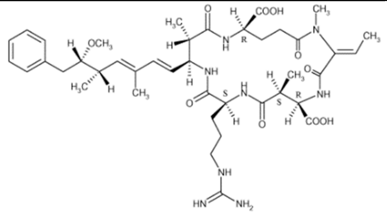	Yes
AP B	$C_{41}H_{60}N_{10}O_{9}$	837.0	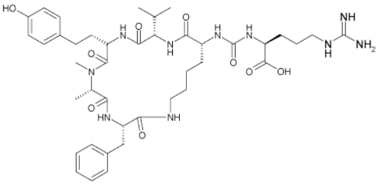	No
AP A	$C_{44}H_{57}N_{7}O_{10}$	844.0	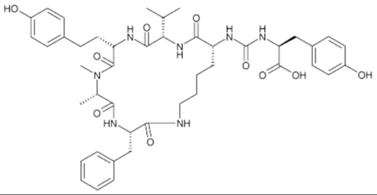	No
MC-LA	$C_{46}H_{67}N_{7}O_{12}$	910.1	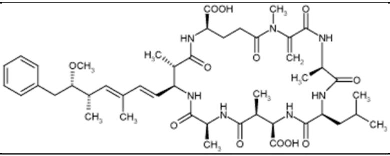	Yes
[D-Asp3]MC-LR	$C_{48}H_{72}N_{10}O_{12}$	981.1	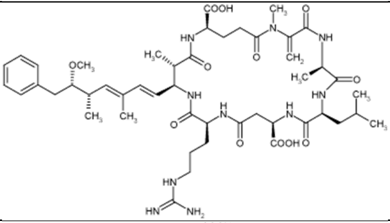	Yes
MC-LF	$C_{52}H_{71}N_{7}O_{12}$	986.2	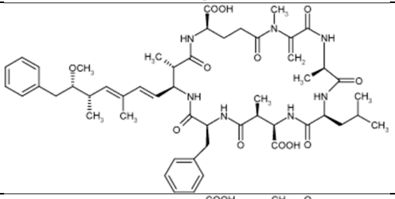	Yes
MC-LR	$C_{49}H_{74}N_{10}O_{12}$	995.2	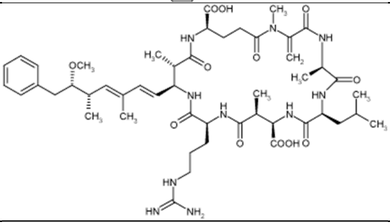	Yes
MC-LY	$C_{52}H_{71}N_{7}O_{13}$	1002.2	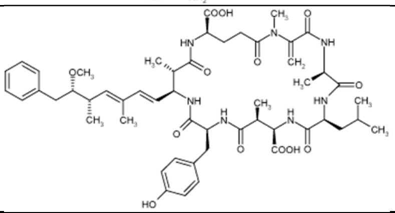	Yes

MC-HilR	$C_{50}H_{76}N_{10}O_{12}$	1009.2	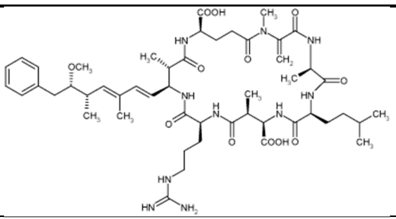	Yes
MC-LW	$C_{54}H_{72}N_{8}O_{12}$	1025.2	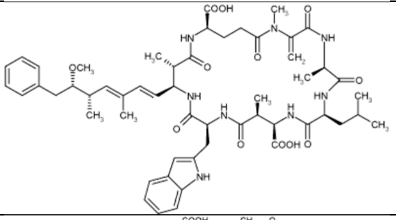	Yes
MC-RR	$C_{49}H_{75}N_{13}O_{12}$	1038.2	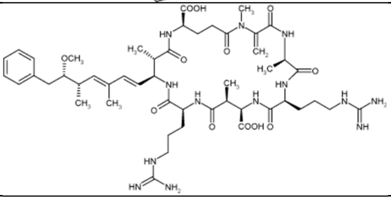	Yes
MC-YR	$C_{52}H_{72}N_{10}O_{13}$	1045.2	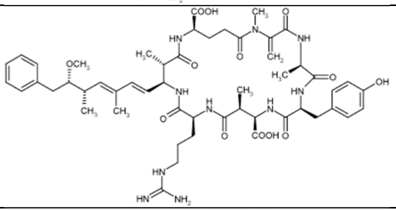	Yes
MC-HtyR	$C_{53}H_{74}N_{10}O_{13}$	1059.2	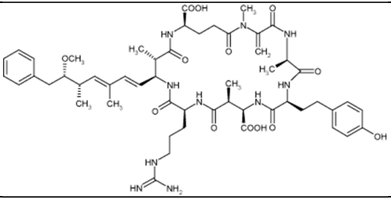	Yes
MC-WR	$C_{54}H_{73}N_{11}O_{12}$	1068.3	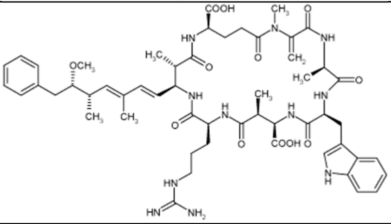	Yes

Table S2. Position 2 and 4 substituents of the MC congeners.

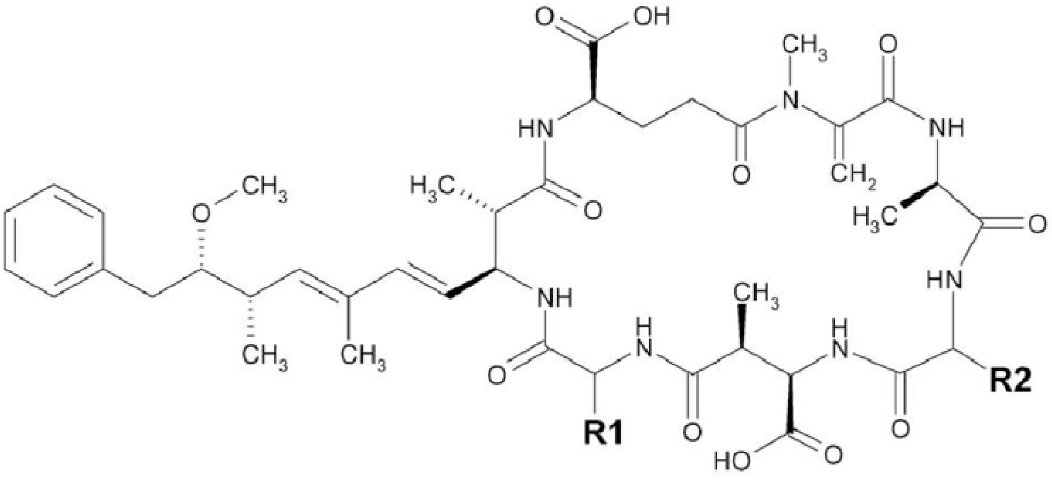

Analyte	Position 2 (R2)	Position 4 (R1)
MC-LR	Leucine	Arginine
MC-RR	Arginine	Arginine
MC-LA	Leucine	Alanine
MC-LY	Leucine	Tyrosine
MC-LF	Leucine	Phenylalanine
MC-LW	Leucine	Tryptophan
MC-YR	Tyrosine	Arginine
MC-WR	Tryptophan	Arginine
MC-HilR	Homoisoleucine	Arginine
MC-HtyR	Homotyrosine	Arginine
[D-Asp3] MC-LR	Leucine	Arginine

Table S3. Selected Ion Monitoring (SIM) method.

Analyte	Precursor Ion (*m*/*z*)	Molecular Ion
MCLR_2H	498.4	$[M + 2H]^{2+}$
MCLR_H	995.0	$[M + H]^{+}$
NOD	825.6	$[M + H]^{+}$
MCRR_2H	519.9	$[M + 2H]^{2+}$
MCRR_H	1038.2	$[M + H]^{+}$
[D-Asp3]MCLR_2H	491.2	$[M + 2H]^{2+}$
[D-Asp3]MCLR_H	981.5	$[M + H]^{+}$
MCWR_2H	534.7	$[M + 2H]^{2+}$
MCWR_H	1068.3	$[M + H]^{+}$
MCWR_2H_Na	545.7	$[M + H + Na]^{+}$
MCLY_2H	501.6	$[M + 2H]^{2+}$
MCLY_H	1002.4	$[M + H]^{+}$
MCLY_2H_NH_4	510.2	$[M + 2H + NH_4]^{2+}$
MCLW_2H	513.2	$[M + 2H]^{2+}$
MCLW_H	1025.4	$[M + H]^{+}$
MCLF_2H	493.6	$[M + 2H]^{2+}$
MCLF_H	986.4	$[M + H]^{+}$
MCHtyR_2H	530.2	$[M + 2H]^{2+}$
MCHtyR_H	1060.4	$[M + H]^{+}$
AP B_2H	419.2	$[M + 2H]^{2+}$

AP B_H	837.4	$[M + H]^{+}$
AP A	845.3	$[M + H]^{+}$
MCYR_2H	523.2	$[M + 2H]^{2+}$
MCYR_H	1045.4	$[M + H]^{+}$
MCHilR_2H	505.2	$[M + 2H]^{2+}$
MCHilR_H	1009.4	$[M + H]^{+}$
MCLA_2H	455.6	$[M + 2H]^{2+}$
MCLA_H	910.4	$[M + H]^{+}$

Table S4. MS/MS parameters and monitored transitions of the analytes studied.

Compound Name	Molecular Ion	Precursor Ion (*m*/*z*)	Product Ion (*m*/*z*)	Dwell (ms)	Collision Energy (V)	Cell Accelerator Voltage (V)	Time segment (min)
MCRR_Q	$[M + 2H]^{2+}$	519.9	134.9	100	30	5	0.10 to 5.40
MCRR_I2	$[M + 2H]^{2+}$	519.9	126.8	100	50	6	
MCRR_I1	$[M + 2H]^{2+}$	519.9	102.8	100	55	6	
AP B_Q	$[M + 2H]^{2+}$	419.2	200.9	100	30	5	
AP B_I2	$[M + 2H]^{2+}$	419.2	70.0	100	55	5	
AP B_I1	$[M + 2H]^{2+}$	419.2	58.0	100	55	5	
NOD_I2	$[M + H]^{+}$	825.6	226.8	20	55	5	5.40 to 5.95
NOD_Q	$[M + H]^{+}$	825.6	134.9	20	70	5	
NOD_I1	$[M + H]^{+}$	825.6	69.9	20	75	5	
MCWR_I1	$[M + 2H]^{2+}$	534.7	518.4	35	5	5	
MCWR_Q	$[M + 2H]^{2+}$	534.7	134.9	35	10	5	
MCWR_I2	$[M + 2H]^{2+}$	534.7	103.0	35	60	5	
MCHtyR_I2	$[M + 2H]^{2+}$	530.2	925.3	10	10	5	
MCHtyR_I1	$[M + 2H]^{2+}$	530.2	513.9	10	5	5	
MCHtyR_Q	$[M + 2H]^{2+}$	530.2	134.9	10	5	5	
MCYR_I1	$[M + 2H]^{2+}$	523.2	507.1	10	5	5	
MCYR_I2	$[M + 2H]^{2+}$	523.2	136.0	10	40	5	
MCYR_Q	$[M + 2H]^{2+}$	523.2	135.0	10	10	5	
MCHilR_I1	$[M + 2H]^{2+}$	505.2	875.3	15	5	5	
MCHilR_I2	$[M + 2H]^{2+}$	505.2	489.1	15	5	5	
MCHilR_Q	$[M + 2H]^{2+}$	505.2	134.9	15	5	5	
MCLR_Q	$[M + 2H]^{2+}$	498.4	134.9	20	12	6	
MCLR_I2	$[M + 2H]^{2+}$	498.4	102.9	20	40	4	
MCLR_I1	$[M + 2H]^{2+}$	498.4	85.8	20	35	5	
[D-Asp3]MCLR_I1	$[M + 2H]^{2+}$	491.2	847.3	20	10	5	
[D-Asp3]MCLR_Q	$[M + 2H]^{2+}$	491.2	134.9	20	15	5	
[D-Asp3]MCLR_I2	$[M + 2H]^{2+}$	491.2	102.8	20	40	5	
MCLW_Q	$[M + 2H]^{2+}$	513.2	135.0	40	10	5	5.95 to 7.50
MCLW_I2	$[M + 2H]^{2+}$	513.2	104.6	40	40	5	
MCLW_I1	$[M + 2H]^{2+}$	513.2	102.9	40	55	5	
MCLY_I1_NH_4 adduct	$[M + 2H + NH_4]^{2+}$	510.2	485.5	45	5	5	
MCLY_Q_NH_4 adduct	$[M + 2H + NH_4]^{2+}$	510.2	134.9	45	5	5	
MCLY_I2_NH_4 adduct	$[M + 2H + NH_4]^{2+}$	510.2	102.8	45	60	5	
MCLF_I2	$[M + 2H]^{2+}$	493.6	852.3	60	5	5	

MCLF_Q	[M + 2H]$^{2+}$	493.6	134.8	60	5	5
MCLF_I1	[M + 2H]$^{2+}$	493.6	85.9	60	35	5
MCLA_I1	[M + 2H]$^{2+}$	455.6	776.2	20	5	5
MCLA_Q	[M + 2H]$^{2+}$	455.6	134.9	20	5	5
MCLA_I2	[M + 2H]$^{2+}$	455.6	102.8	20	45	5
AP A_I1	[M + H]$^{+}$	845.3	58.0	60	75	5
AP A_Q	[M + H]$^{+}$	845.3	84.0	60	40	5
AP A_I2	[M + H]$^{+}$	845.3	638.1	60	25	5

Table S5. Thermal spring water samples properties.

Location	pH		Conductivity (mS/cm)		Temperature (ºC)		Classification*	Sulphure content (mg/L)
	June	September	June	September	June	September		
Zújar	7.54	7.20	6.16	6.24	39.00	39.50	mesothermal	<0.05
Dúrcal	7.65	7.73	0.95	1.16	25.00	24.80	hipothermal	<0.05
Santa Fe	7.47	7.46	3.43	3.39	40.30	39.20	mesothermal	<0.05
La Malahá	7.57		2.47		28.50		hipothermal	<0.05
Alicún de las Torres	7.44		1.98		34.00		hipothermal	<0.05
Alhama de Granada	8.24		0.69		37.60		mesothermal	<0.05

* Cold <20ºC; Hypothermal 21-35 ºC; Mesothermal 35-45 ºC; Hyperthermal >45 ºC.

Table S6. Matrix Effect and Recovery assays from ZÚJAR thermal water samples.

Analytes	Recoveries (%)				Matrix Effect (ME)			
	L1	RSD (%) (L1)	L2	RSD (%) (L2)	L1	RSD (%) (L1)	L2	RSD (%) (L2)
**MCRR**	89.67	3.02	84.28	4.03	17.69	6.19	-0.88	5.79
**AP B**	89.73	8.59	86.97	4.44	1.53	7.68	-6.89	2.81
**MCWR**	84.92	3.14	85.01	2.25	-2.61	1.76	-9.68	2.71
**MCLR**	88.97	3.57	98.35	8.41	2.19	4.48	-10.59	7.27
**MCHilR**	87.59	6.51	104.48	7.56	14.64	2.74	-18.22	4.30
**[D-Asp3]MCLR**	105.86	5.82	92.22	3.88	-1.16	6.40	-3.71	3.92
**NOD**	86.67	2.81	82.39	3.91	9.89	3.18	-6.03	5.27
**MCHtyR**	92.44	9.30	84.55	7.27	2.83	9.16	-17.13	7.25
**MCYR**	89.46	14.36	82.06	3.52	1.33	15.92	-1.02	4.11
**AP A**	94.55	5.85	87.17	3.87	0.66	3.50	12.07	5.17
**MCLW**	81.42	6.59	80.79	5.27	-12.60	7.39	-12.18	6.35
**MCLF**	92.26	7.83	83.52	2.55	-12.04	5.02	-15.03	4.94
**MCLA**	82.01	4.45	99.44	6.22	-6.54	8.51	-10.93	12.32
**MCLY**	96.85	5.44	85.19	4.48	-3.69	3.85	-16.42	1.70

* Concentration levels. L1 and L2, respectively: [D-Asp3]MCLR and MCYR 0.04 and 0.16 μg/L; AP B, MCHilR, MCRR, MCHtyR, MCLW 0.04 and 0.32 μg/L; MCLR, MCWR, MCLA 0.12 and 0.48 μg/L; MCLY, NOD, AP A and MCLF 0.02 and 0.80 μg/L.

Table S7. Matrix Effect and Recovery assays from DURCAL thermal water samples.

Analytes	Recoveries (%)				Matrix Effect (ME)			
	L1	RSD (%) (L1)	L2	RSD (%) (L2)	L1	RSD (%) (L1)	L2	RSD (%) (L2)

**MCRR**	81.20	2.80	80.21	4.60	16.16	5.90	4.67	5.38
**AP B**	106.91	6.75	97.37	8.91	6.17	8.98	62.04	7.31
**MCWR**	92.61	1.52	86.76	3.65	2.03	2.06	-18.70	5.92
**MCLR**	101.94	3.83	89.71	7.62	-7.33	2.78	-11.90	5.88
**MCHilR**	98.04	3.36	87.80	1.31	11.35	1.52	-10.06	3.07
**[D-Asp3]MCLR**	104.31	2.23	87.54	5.03	-16.06	2.94	-4.70	4.68
**NOD**	94.27	4.07	97.48	6.57	-4.03	3.81	-6.89	8.50
**MCHtyR**	101.43	8.51	86.59	6.13	-1.41	2.30	-8.64	7.43
**MCYR**	93.87	5.11	101.72	7.06	15.49	7.39	-18.97	7.41
**AP A**	104.79	4.18	96.60	2.96	-3.95	6.68	-2.91	4.23
**MCLW**	93.85	7.12	84.89	5.48	-7.76	1.52	-12.52	3.70
**MCLF**	89.10	6.12	88.31	5.69	-18.32	5.58	-16.39	6.13
**MCLA**	95.39	2.39	91.61	10.04	-17.84	2.63	-13.34	9.30
**MCLY**	88.26	7.44	84.13	4.17	-0.22	2.19	-10.22	1.15

* Concentration levels. L1 and L2, respectively: [D-Asp3]MCLR and MCYR 0.04 and 0.16 µg/L; AP B, MCHilR, MCRR, MCHtyR, MCLW 0.04 and 0.32 µg/L; MCLR, MCWR, MCLA 0.12 and 0.48 µg/L; MCLY, NOD, AP A and MCLF 0.02 and 0.80 µg/L.

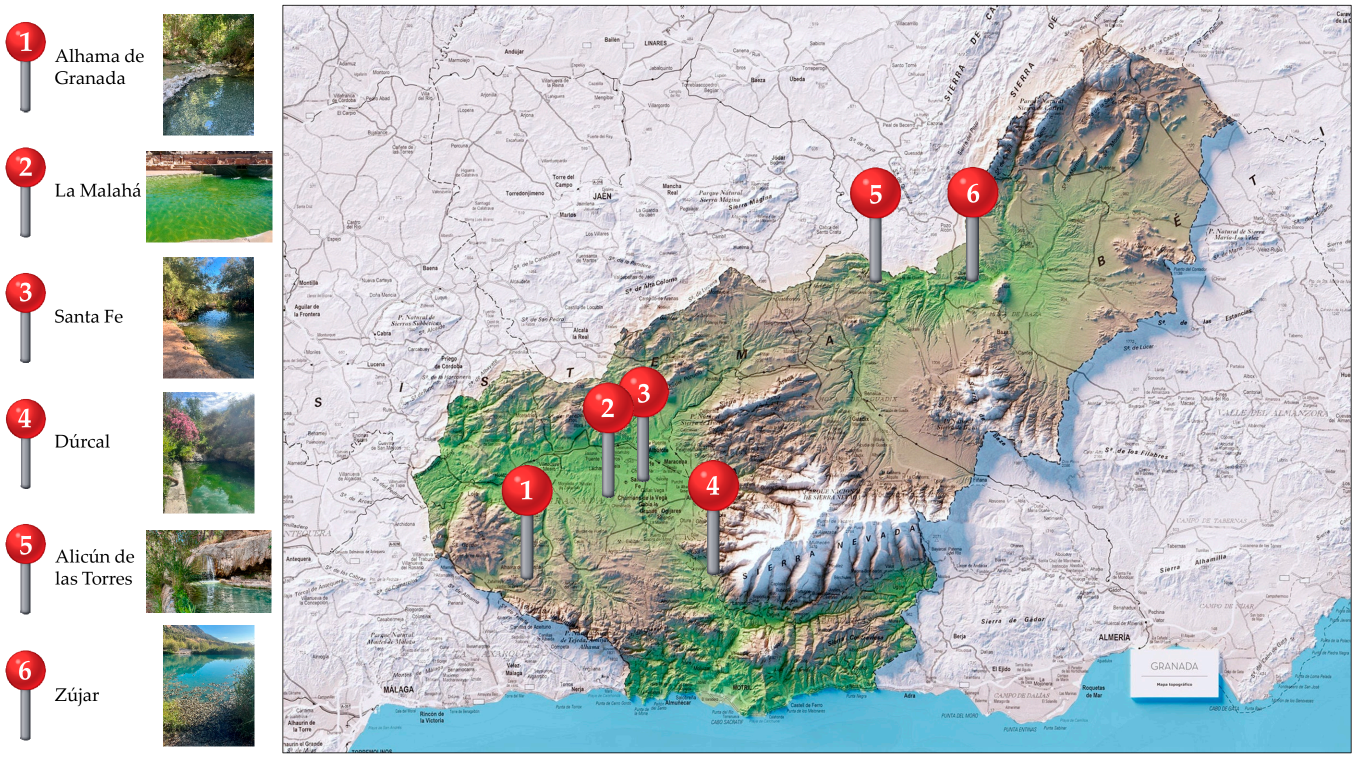

Figure S1. Location of sampling points in the province of Granada (Andalucía, Spain).

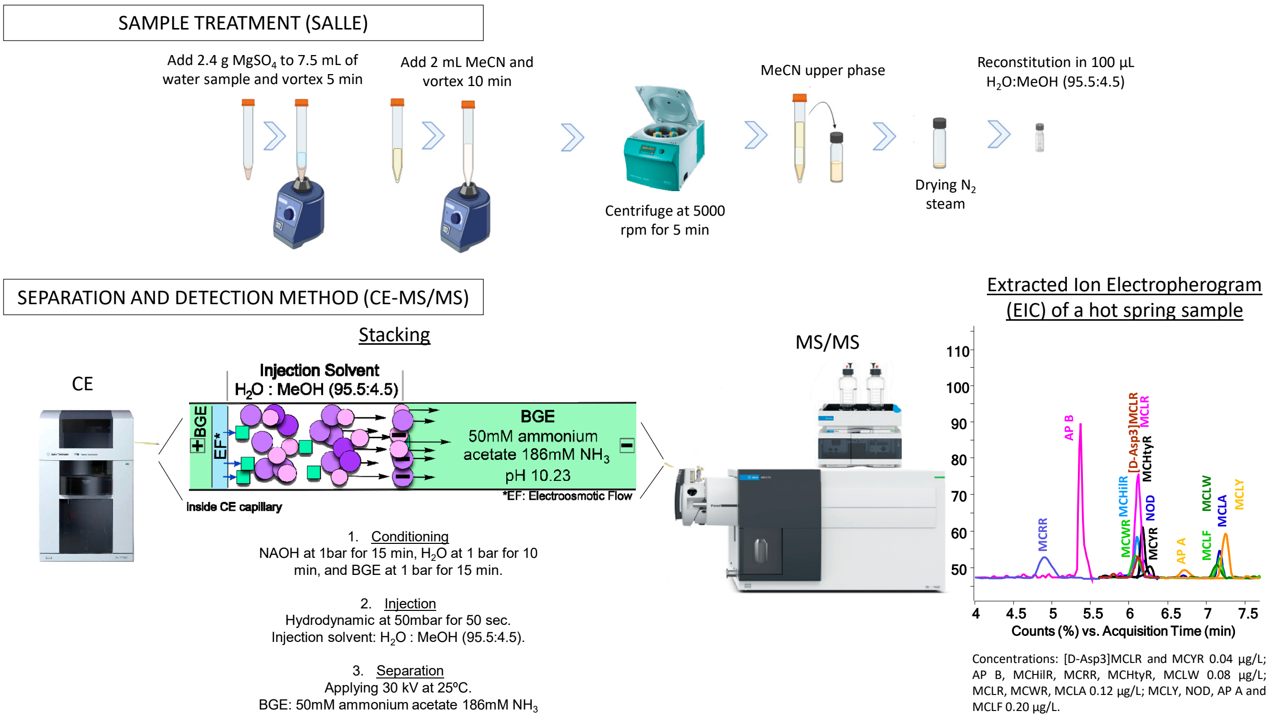

Figure S2. Schematic diagram of the SALLE-CZE-MS/MS method.
